# Chemical analysis and sensory evaluation of honey produced by honeybee colonies fed with different sugar pastes

**DOI:** 10.1002/fsn3.1843

**Published:** 2020-09-24

**Authors:** Bouchra Moumeh, María Dolores Garrido, Pedro Diaz, Irene Peñaranda, María Belén Linares

**Affiliations:** ^1^ Department of Food Science and Technology, Veterinary Faculty University of Murcia Murcia Spain

**Keywords:** feeding, honey bee, protein, sensory analysis, sugar paste

## Abstract

Supplemental feeding of honeybee (*Apis mellifera L*.) colonies is essential for colony buildup. Honey samples obtained from feeding honey bee colonies with different commercial sugars, including sugar paste, sugar paste + vitamins +amino acids, and sugar paste + vitamins +protein as pollen substitute, were studied to determine the effects of feeding bees on the physicochemical and sensory characteristics of honey, compared with the honey produced by a control group (no supplemental feeding). Analyzed honey samples from the different groups were in accordance with the criteria described in Council directive 2001/110/CE, 2002. Elsewhere, significant differences (*p* > .05) were detected in color, free acidity, diastase activity, hydroxymethylfurfural, sugar profile, and conductivity between all honey. In terms of mineral content, the honey from hives faded with sugar paste + vitamins +protein and control group had higher values for Na, Mg, P, K, Ca, Mn, Fe, Cu, and Zn. Related to sensory analyses, no differences in flavor and basic taste were found in all analyzed honey (*p* > .05) independently the type of feeding. For the visual attributes, only differences were found for the color. Supplementary feeding with different sugar pastes and proteins does not affect the physicochemical characteristics of honey. For the sensory analyses, control sample presented significant differences only for color and chemical odor attributes compared with honey from bees receiving supplementation.

## Graphical abstract

This paper is based on the effect of feeding supplementation of honeybee and the effect on honey quality.

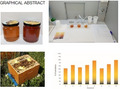

## 1 INTRODUCTION

Honey is a viscous, aromatic, sweet food that is consumed and enjoyed by people all around the world. For this reason, it requires certain standards and norms that guarantee its identity and quality so that consumers feel they can safely consume it, and at the same time, the product can enjoy circulation in the internal market and access to the external market “Council directive, 2001/110/RC, 2002.”

Many regions of the world have recorded honeybee losses in recent years. The cause of the decline in bee numbers has not been identified, although there are several factors, acting in combination or separately. These are thought to include climatologically difficult years with a consequent nutritional impact on colonies, the effect of neonicotinoid insecticides and unsuitable management practices (Pajuelo, Torres, & Bermejo, 2008).

The nutrition of bees is essential in certain periods of the year (Guler et al., 2014). Supplementary feeding can range from a diet that consists almost exclusively of carbohydrates, to others more balanced that carry energy, protein, and lipid. In the first case, the main interest is to accelerate the energetic metabolism of the whole hive, while in the second case it is sought to stimulate breeding and so increase in population density. In general, honeybees feed upon nectar of flowers and pollen. The nectar fulfills their carbohydrate requirements (Brodschneider & Crailsheim, 2010). However, the use of pollen as a protein and lipid source has its contraindications, since pollen can be the vehicle for transmission of pathogens (Durrer & Schmid‐Hempel, 1994; Singh & Kundu, 2010).

The carbohydrate needs of honeybee colonies can be provided by sugar cane, sugar beet, and sugar maize due to their low cost (Ruiz‐Matute, Weiss, Sammataro, Finely, & Sanz, 2010; Sammataro & Weiss, 2013). Certainly, supplementary diets with increased protein intake have been provided for decades (Standifer, Moeller, Kauffeld, Herbert, & Shimanuki, 1978; Herbert, 1992.

Of great importance the adequate feeding during of seasons when food resources are drastically reduced (such as winter or dry periods), with little natural food available to maintain the queen during egg‐laying and to generate healthy of spring, renew food stocks and allow honey production (DeGrandi‐Hoffman et al., 2016; Morais et al., 2013). The effect on the quality of honey of supplementing the bee diet with protein has not been studied, but it is known that feeding bees with high percentages of sucrose, corn syrup, and high fructose syrup can generate problems of indirect adulteration of the honey obtained (Guler, Bakan, Nisbet, & Yavuz, 2007). The effect of supplementation on the physicochemical and the sensory quality of honey has not studied in detail.

The sensory analysis applied to honey is an important complement to the physico‐chemical parameters and pollen analyses. It can confirm defects in fermentation, and the presence of impurities, the odor of smoke, metallic tastes, and other characteristics that common laboratory routine analyses do not access (Piana et al., 2004). In this sense, many studies have been carried out on physicochemical and sensory analyses for use as analytical tools for the quality control of honey in relation to its botanic origin (Anklam, 1998; Galán‐Soldevilla, Ruiz‐Pérez‐Cacho, Serrano, Jodral, & Bentabol, 2005; González‐Lorente, De Lorenzo, & Pérez‐Martin, 2008).

The present study aimed to evaluate the physicochemical compositions and sensory properties of the honey produced by honeybees fed with supplemental sugars pastes of different compositions, including vitamins, amino acids and/or protein as pollen substitute, compared with the honey produced by a control group (with no supplemental feeding).

## 2 MATERIALS AND METHODS

### 2.1 Supplemental Feeding and honey harvest

The feeding and harvest were carried out in the beekeeping unit of Research Farm of the University of Murcia, Faculty of Veterinary, starting the experimental treatments in December 2017. Twenty four colonies were distributed randomly into four experimental groups with various feeding supplements: M1, control group (no supplemental feeding); M2 sugar paste; M3 sugar paste with vitamins and amino acids; M4, sugar paste with 3% protein (pollen substitute) and vitamins. The colonies received the supplements in plastic trays containing 1 kg of the supplement every 15 days during the experimental period (from December 2017 to April 2018).

The honey was collected in April 2018 by centrifugation and filtered through a sieve. Honey obtained was kept unpasteurized in glass containers at room temperature until physicochemical and sensory analysis.

### 2.2 Analytical procedures

#### 2.2.1 Chemical analyses

The water content was determined using a refractometer at 20ºC. The samples were homogenized at room temperature and directly deposited in the prism of the refractometer. The obtained refractive index in each sample was related to the water content of the honey, according to the relationship between honey water content and refractive index (Bogdanov, Ruoff, & Oddo, 2004; Chataway, 1932).

The pH was measured directly in the water solution of the honey sample using a pH‐meter (CRISON, GLP 21) (AOAC, 2012; Bogdanov, 2009). The electrical conductivity was measured in accordance with AOAC (2009). Twenty grams of honey in distilled water were weighed and transferred to a 100 ml flask, completing the volume with water. The solution was transferred to a beaker, and the electrodes were immersed. The reading of the conductance of the solution was in µs/cm.

Hydroxymethylfurfural (HMF) was determined by using the AOAC (1990) Official Method 980.23. Five grams of honey was dissolved in 25 ml of distilled water and treated with a clarifying agent (0.5 ml of Carrez I and 0.5 ml of Carrez II solutions), and the volume was made up to 50 ml. The solution was filtered, and the first 10 ml was discarded. The absorbance of the filtered solution was measured at 284 and 336 nm against an aliquot of the filtered solution treated with NaHSO_3_. HMF was determined as follows: HMF/100 g of honey = (Abs 284–Abs 336) × 14.97 × (5/g of the sample).

The diastase activity was measured using the Phadebas method for α‐amylase. Phadebas is a synthetic reagent that produces a blue color when it is hydrolyzed by the diastase. Adsorption was determined using a UV/VIS spectrophotometer at 620 nm, the absorbance is directly proportional to the diastase activity in the honey sample. Results were expressed in Gothe units per gram of honey (Bogdanov, 2009).

The sugar content was determined by HPLC with a RI (refractive index) detector and analytical stainless‐steel column in polar aminopropylsilane (NH2). Five grams of honey dissolved in water were transferred to a 100 ml volumetric flask containing 25 ml of methanol and topped up with water. The solution was filtered through a syringe filter (Bogdanov, 1997).

The color was measured according to the Pfund color scale according to Council directive, 2001/110/EC; the reading is expressed in millimeters.

Ash content was determined according to the AOAC (2009) method. The minerals content was analyzed using an Inductively Coupled Plasma Mass Spectrometer (Varian Spectraa‐600; Agilent Technologies, Santa Clara, CA, USA). In a preliminary step, samples were heated and sonicated to facilitate honey homogenization. Aliquots of 0.5 g honey were digested in a microwave oven using 5 ml of HNO_3_ + H_2_O_2_. The following minerals were quantified: K, Ca, P, Na, Mg, Fe, Zn, Cu, and Mn (Caroli, Forte, Iamiceli, & Galoppi, 1999).

### 2.3 Sensory evaluation

#### 2.3.1 Panel selection

Eight panelists aged from 20 to 52 from the Faculty of Veterinary, University of Murcia, previously selected and trained according to ISO 8,586, were further trained in the appearance, odor, flavor, taste, mouthfeel, and textural parameters of honey (Table 1). All the sensory analyses were carried out following Piana et al. (2004) and the norms of ISO 8589:^2007^; ISO 8586–2:^2008^; ISO 8586:^2012^.

**TABLE 1:** Attributes considered for honey sensory description

Attributes	Description
Visual attributes	
Color intensity	Degree of amber color (varying from water white to dark ambar)
Crystallization	Phenomenon that causes the loss of fluidity. The size of the crystals must be uniform (for crystallized honey).
Viscosity	Force required to remove honey from a spoon (for liquid honey)
Olfactory and aroma attributes	
Overall intensity	Strength of the stimuli perceived by the nose or by olfactory receptors via retronasal way.
Floral	Associated with different flowers
Fruity	Associated with different fruits: acid, ripe and tropical
Vegetal	Associated with gardens, green notes, dry leaves, and wood
Warm	Associated with foods characterized by their sweet smell and taste.
Chemical	Not associated with food, it is characterized by its aggressiveness (smoked, phenolic, sulfuric, vinegary).
Animal	Associated with animals and/ or degradation (mold, urine, stable...)
Taste and Mouthfeel	
Sweetness	Sensation produced by products that contain sugars such as sucrose and fructose.
Sourness	Sensation produced by products that contain acids, such as citrus.
Saltiness	Sensation produced by products that contain salts, such as sodium chloride
Bitterness	Sensations produced by products such as caffeine.
Persistence	Feeling similar to what is perceived while the product was in the mouth and while continuing over a period of time measurable.
Astringency	Organoleptic property of pure substances or mixture which produce an astringent sensation.
Freshing sensation	Sensation of freshness in the oral cavity (similar to that produced by mint)
Texture attributes	
Adhesiveness	Ability of honey to stick to the teeth and oral cavity.
Granularity	Geometric attribute of texture relative to the perception of the size and shape of the particles in crystalline honey.

### 2.4 Sample preparation and testing procedure

Six samples from each of the four groups (M1, M2, M3, and M4) were presented to the tasting panel. The samples of honey were presented in transparent 100 ml jars containing 30 g of honey at 20 ± 2ºC. Each sample was given a random three‐digit code and presented in a different order for each panelist sample was evaluated in triplicate in different sessions, in which three or four samples were presented.

Assessors made a descriptive quantitative analysis of kinds of honey using an unstructured scale (10 cm) by evaluating the appearance (color, fluidity, and the crystallization) and olfactory analysis (odor intensity). The first odor contact could be reinforced by extending the honey sample on the container walls with a spatula. Besides, each taster had a bottle with coffee beans in order to relax the smell. For basic tastes and aroma evaluation, a small amount of honey was placed on the tongue with a disposable spatula. The sample was allowed to dissolve for a few seconds while the subject did not inhale. Air was released through the nose, keeping the mouth closed, so that the aromas stimulate the olfactory receptors. Water and low salt bread were provided to clean the palate between samples. Finally, texture attributes (adhesiveness and crystallization) were determined.

#### 2.4.1 Statistical analysis

The statistical analysis used IBM SPSS STATISTIC (Version 24). An analysis of variance (ANOVA) and Tukey's HSD multiple comparison tests (*p* < .05) was carried out to establish the difference between pairs of groups, according to the physicochemical and sensory parameters.

## 3 RESULTS AND DISCUSSION

### 3.1 Chemical analyses

The water content is one of the most important characteristics influencing the physical properties of honey (Escuredo, Míguez, Fernández‐González, & Seijo, 2013). The samples from the present study did not present significant differences (*p* > .05) concerning the type of supplement administered to bees (Table 2). The water content values are within the range found in Greek honey (10.50% −20.50%) (Karabagias, Badeka, Kontakos, Karabournioti, & Kontominas, 2014) and are lower than those obtained in blossom honey from Spain (15.50%–18.90%) (Bentabol, Hernández, Rodríguez, Rodríguez, & Díaz, 2014). In general, the values for all the honey samples were within the established legal requirements (Council Directive, 2001).

**TABLE 2:** The means and standard deviations of physicochemical parameters

Attributes/Samples	M1	M2	M3	M4
	*M* ± *SD*	*M* ± *SD*	*M* ± *SD*	*M* ± *SD*
Water content (%)	13.00 ± 0.00	13.20 ± 0.00	13.20 ± 0.00	13.40 ± 0.00
pH	3.31 ± 0.09	3.32 ± 0.00	3.28 ± 0.00	3.29 ± 0.00
Free acidity meq/kg	16.83 ± 0.28^b^	15.33 ± 0.57^a^	14.68 ± 0.29^a^	14.17 ± 0.28^a^
Color (mm Pfund)	32.48 ± 1.19^a^	46.84 ± 1.36^c^	39.17 ± 2.52^b^	28.40 ± 0.56^a^

Color description	White	Extra light amber	Extra light amber	White
Diastase activity (Gothe scale)	20.86 ± 0.06^d^	22.75 ± 0.38^e^	22.76 ± 0.81^e^	23.11 ± 0.15^e^
Hydroxymethylfurfural (mg/kg)	4.76 ± 0.08^b^	2.55 ± 0.00^a^	2.57 ± 0.00^a^	2.81 ± 0.21^a^
Conductivity (µs/cm)	300 ± 0.00^b^	363.30 ± 3.74^c^	256.67 ± 3.70^a^	456.67 ± 4.97^d^
Ash content %	0.24 ± 0.02^b^	0.30 ± 0.01^c^	0.20 ± 0.01^a^	0.37 ± 0.03^d^

Note

M1: honey from non‐supplemented bees; M2: honey from supplemented bees with sugar; M3: honey from supplemented bees with sugar + vitamins+amino acids; M4: honey from supplemented bees with sugar + 3% of protein. Values within rows with different letters differ significantly (*p* > .05).

A pH level of between 3.2 and 4.5 and the natural acidity of honey inhibit the growth of microorganisms (Karabagias et al., 2014). The type of supplementation used did not alter the pH value, and the pH range was within that accepted for honey (Bogdanov et al., 2004). Values for free acidity ranged from 14.17 to 16.83 meq/kg. Differences between acidity values may be the result of floral origin, the presence of organic acids, or the geographical origin (Isla et al., 2011). In our analysis, there were significant differences (*p* > .05) between the honey from the control group and all kinds of honey from supplemented groups, regardless of the type of supplementation. However, all the values for free acidity were below the maximum levels (50 meq/kg) (Council Directive, 2001).

The color ranged from 28.40 to 46.84, according to the Pfund scale. Control honey and honey from bees receiving the sugar paste supplement had lower color values (32.48 and 28.40, respectively), there are no studies that analyze the effect of supplementation on the color of honey. In general, color values depend on the mineral content and floral origin (Piana et al., 2004), but the qualitative pollen analysis of our samples showed that the diversity of pollen types was similar between honey groups. Hence, the difference in color between the honey samples would have been mainly due to the supplementation administered, since the botanical origin was the same for all the groups.

Diastase is a natural honey enzyme and its activity is used in Europe as a determinant of freshness. Table 2 illustrates diastase activity values ranged from 20.86 to 23.11 (Gothe scale). Independently of the type of supplementation received by the honeybees a significant statistical difference (*p* > .05) was found between control honey (M1) and the other kinds of honey (M2, M3, M4). The lowest value was found in control honey while the highest was detected in the sugar paste + 3% of protein honey. However, all samples studied conform to the required standards (Council Directive, 2001).

The HMF level is not only indicative of honey freshness but also of storage duration and conditions (Shapla, Solayman, Alam, Khalil, & Gan, 2018). All the analyzed samples can be considered fresh, presenting levels below the maximum established by international standards (<40 mg/kg) (Council Directive, 2001). Significant differences (*p* > .05) were found between control honey and the rest of the samples. The HMF values in our study ranged from 2.55 to 4.76 mg/kg, the highest value referring to the control of honey. These values fall within the range found for different blossom honey from Spain (0.70–26.00) (Bentabol et al., 2014).

The ash content of the honey samples, determining the mineral richness and the resulting electrical conductivity. These parameters are important for establishing the botanical origin of a honey sample as differentiating features between nectar and honeydew honeys (Aazza, Lyoussi, Antunes, & Miguel, 2014; Louveaux, 1959). Statistically significant differences (*p* > .05) were found between all honey samples. The ash content was between 0.20% (M2) and 0.37% (M4), both values below the maximum established for honey 0.6%. The electrical conductivity values of the honey samples varied between 256.67μS/cm and 456.67μS/cm (Table 2). The electrical conductivity of honey is related to the ash content and acidity (Yücel & Sultanog, 2013), and should not surpass 800 μS/cm in blossom honey, from a quality control point of view (Kaškonienė, Venskutonis, & Čeksterytė, 2010).

In terms of mineral content, significant differences (*p* < .05) were found in our samples (Table 3). Potassium was the predominant mineral in all the samples, ranging from 215.85 (M3) to 418.51 mg/L (M3). M4 was richer in sodium and calcium than the rest of the samples. While iron was not detected in M1 and M3 samples, other samples contained concentrations ranging from 0.07 (M2) and 0.65 mg/L (M4). The concentration of copper ranged between 0.12 (M2, M3) and 0.20 mg/L (M4). The mineral content of the honey varied greatly, as previously observed in honey from Spain, and Portugal (Escuredo et al., 2013; Silva, Videira, Monteiro, Valentão, & Andrade, 2009).

**TABLE 3:** Mineral composition (mg/l) (average ± *SD*) of the different types of honeys

Mineral/Sample	M1	M2	M3	M4
Sodium (Na)	26.90 ± 0.28^b^	11.44 ± 0.35^a^	10.81 ± 0.02^a^	35.69 ± 0.05^c^
Magnesium (Mg)	12.91 ± 0.61^b^	5.86 ± 0.11^a^	5.42 ± 0.12^a^	6.64 ± 0.01^c^
Phosphorus (P)	58.01 ± 0.89^b^	34.90 ± 0.36^a^	34.75 ± 0.06^a^	38.95 ± 0.02^c^
Potassium (K)	215.85 ± 1.45^b^	272.30 ± 1.44^a^	418.51 ± 1.03^c^	277.71 ± 1.08^d^
Calcium (Ca)	38.92 ± 0.63^c^	32.04 ± 0.92^a^	29.00 ± 0.73^b^	47.26 ± 0.87^d^
Manganese (Mn)	0.120 ± 0.01^c^	0.061 ± 0.00^a,b^	0.037 ± 0.00^a^	0.093 ± 0.00^b,c^
Iron (Fe)	0.00 ± 0.00^a^	0.07 ± 0.00^b^	0.00 ± 0.00^a^	0.65 ± 0.00^c^
Nickel (Ni)	0.02 ± 0.00^b^	0.02 ± 0.00^a,b^	0.01 ± 0.00^a^	0.02 ± 0.00^a,b^
Copper (Cu)	0.13 ± 0.11^a^	0.12 ± 0.00^b,a^	0.12 ± 0.00^a^	0.20 ± 0.00^b^
Zinc (Zn)	0.67 ± 0.08^c^	0.36 ± 0.05^b^	0.27 ± 0.07^a^	0.91 ± 0.00^d^

Note

M1: honey from non‐supplemented bees; M2: honey from supplemented bees with sugar; M3: honey from supplemented bees with sugar + vitamins+amino acids; M4: honey from supplemented bees with sugar + 3% of protein. Values within rows with different letters differ significantly (*p* < .05).

### 3.2 Sugar composition and Granulation ratios

Sugars in honey depend mostly on the botanical and geographical origins, although other factors, such as the weather, processing, and storage conditions, may also intervene (Dobre, Georgescu, Alexe, Escuredo, & Seijo, 2012; Escuredo, Dobre, Fernández‐González, & Seijo, 2014; Tornuk et al., 2013).

The analysis of the sugar composition of studied honey is shown in Table 4. Significant statistical differences were found for the sugar composition between all the honey groups evaluated (*p* < .05). The major carbohydrates in the honey were fructose and glucose.

**TABLE 4:** Sugar composition and granulation indexes (average ± *SD*) for the different types of honeys

Attributes/Samples	M1	M2	M3	M4
Sugar Composition				
Fructose (%)	50.17 ± 0.08^a^	53.26 ± 0.27^d^	50.69 ± 0.33^a,b^	53.29 ± 0.03^d^
Glucose (%)	40.96 ± 0.14^b^	41.88 ± 0.23^c^	40.41 ± 0.40^b^	39.61 ± 0.13^a^
Sucrose (%)	6.99 ± 0.06^e^	2.99 ± 0.10^b^	7.88 ± 0.04^f^	6.23 ± 0.10^d^
Maltose (%)	1.84 ± 0.16^a,b^	1.91 ± 0.18^b^	0.99 ± 0.77^a,b^	0.87 ± 0.01^a^
Granulation indexes				
F‐G	9.20 ± 0.19^a^	11.38 ± 0.47^c^	10.28 ± 0.7^b^	13.68 ± 0.12^d^
F/G	1.22 ± 0.00^a^	1.27 ± 0.01^b^	1.25 ± 0.02^b^	1.34 ± 0.04^c^
G/W	3.15 ± 0.01^c^	3.17 ± 0.02^c^	3.06 ± 0.03^b^	2.95 ± 0.06^a^
(G‐W)/F	0.56 ± 0.02^c^	0.54 ± 0.02^b^	0.54 ± 0.03^b^	0.50 ± 0.05^a^

Note

M1: honey from non‐supplemented bees; M2: honey from supplemented bees with sugar; M3: honey from supplemented bees with sugar + vitamins+amino acids; M4: honey from supplemented bees with sugar + 3% of protein. Values within rows with different letters differ significantly (*p* < .05).

The honey from bees receiving only sugar paste (M2) and from those receiving sugar paste + 3% of crude protein (M4) had a higher fructose concentration than the no supplemented honey (M1) and the sugar paste + vitamins +free amino acids honey (M3).

In terms of glucose content, M2 had a higher mean value (31.41g/100g) than the other honey (*p* < .05), the lowest mean value corresponding to M4 (29.71g/100g). Honey with a glucose content lower than 30% shows a slow granulation phenomenon over time (Manikis & Thrasivoulou, 2001) so that M2 would show quicker granulation in our study.

Honey from bees receiving sugar paste + vitamins+free amino acids (M3) had the lowest mean sucrose content (2.24 g/100 g), a level significantly different from all the other honey. All the honey samples analyzed in this study did not exceed the limit of 5g/ 100g established by the Council of the European Union. The highest maltose concentrations (1.91%) were found in honey from bees receiving sugar paste alone.

The time honey will take to granulate depends on the Fructose/Glucose (F/G) and the Glucose/Water content (G/W) ratios. Honey with glucose to water ratio of 1.7 or less are considered nongranulating, while honey with ratios of 2.1 or more predicts rapid granulation (Dobre et al., 2012; Kaakeh & Gadelhak, 2005). Similarly, a glucose‐water to fructose ratio higher than 0.50 predicted rapid granulation and a ratio lower than 0.20 predicted slow granulation. The F/G ratio, therefore, is an important parameter to explain honey granulation because glucose is less water‐soluble than fructose, and so induces a tendency to granulation (Escuredo et al., 2014).

Table 4 shows the granulation indices for our honey samples. Honeys M1, M2, M3, and M4 had an F/G ratio of 1.22, 1.27, 1.25, and 1.34, respectively, values which indicated a greater tendency to granulation regardless of the type of supplementation. According to Venir, Spaziani, and Maltini (2010), honey fructose to glucose ratio of 1.14 indicates a tendency to granulate more rapidly than honey with a ratio significantly above 1.58. As observed in previous studies that values above 1.3 had a slower granulation tendency (Dobre, Escuredo, Rodriguez‐Flores, & Seijo, 2014). The F/G ratio of around 1.2 found in our samples was within the range reported in other studies (Bentabol, García, Galdón, Rodríguez, & Romero, 2011; Dobre et al., 2012).

In terms of the glucose to water ratio M1 (2.36), M2 (2.38), M3 (2.29), and elsewhere M4 (2.21) some researchers have indicated that the G/W ratio can be a better indicator for the prediction of honey granulation (Dobre et al., 2012; Manikis & Thrasivoulou, 2001).

According to Dobre et al., honey granulation is slow when the G/W ratio is less than 1.7 and is complete and rapid when the ratio is greater than 2.

All the samples had fructose to glucose proportions greater than 1 (Table 4) regardless of the type of supplementation administrated, which indicates a greater tendency to granulate in these honeys. In terms of the (glucose‐water) to fructose ratio, M1, M2, M3, and M4 indicated a greater tendency to granulation with ratios of 0.47, 0.45, 0.45, and 0.40, respectively. When granulation is incomplete, the crystalline layer is overlaid by a layer of liquid honey with a water content that is higher than that in the original honey. This creates a favorable environment for yeast growth and may lead to fermentation (Escuredo et al., 2013; Tornuk et al., 2013).

### 3.3 Sensory evaluation

In the present study, all honey analyzed were found to have a similar sensory mean value for “Overall odor intensity,” although there was a tendency for this attribute to be higher in the control honey and M3 showed the lowest values (Table 5b). By contrast, there were no significant differences in the floral attribute between honey, although the highest value was observed in the control, honey. Also, all the honey was found to be characterized by fruity, warm, and vegetal attributes. The control honey was distinguished by its aromatic attribute, while this attribute was not perceived in honey from the honey involving supplementation. All honey samples were characterized by the complete absence of the animal attribute. No significant statistical differences in the basic taste were found (*p* < .05) (Table 5a). There were no significant differences in the sensory perception of sweetness, regardless of the types of supplementation, with the highest level of 7 being perceived in the control honey and the lowest level of 5.88 in M3. However, the mean value for the sourness attribute in the honey varied from 0.58 (M3) to 0.13 (M2). The highest mean value of saltiness was 0.41 obtained in the honey involving supplementation with sugar + 3% proteins and the lowest value of 0.30 was perceived in M2 and M3. The bitterness attribute was not perceived in any honey, and neither were chemical and animal attributes. In general, the honey from the control group was sweeter than the honey from supplemented groups. Similarly, the control honey scored better for color, the intensity of odor, and the attributes of smell (Table 5a and 5b). This result agrees with the result of a study of Turkish honey (Guler et al., 2007) from bees that had been fed with sucrose syrup. Honey M4 was classified first for the intensity of aroma, warm, aromatic, and vegetal attributes. Honeys M2 and M3 had an intermediate value between the control honey and honey M4, without having significant statistical differences (*p* < .05), independently if the bees have been supplemented or not. The sensory characteristics of honey depend fundamentally on their botanical origin, so they can vary in smell and taste (Bogdanov et al., 2004). On the other hand, the mineral contents are related to the taste of the honey; the higher the number of minerals, the stronger the flavor (González‐Miret, Terrab, Hernanz, Fernandez‐recamales, & Heredia, 2005). However, the honey from the present study differed from this trend since no significant differences were observed in sensory attributes, although significant differences were recorded in the mineral content (*p* < .05).

**TABLE 5:** (a) Visual, taste, and texture sensory attributes (average ± *SD*) for the honeys. Olfactory and aroma sensory attributes (average ± *SD*) for the different honeys (b)

Attributes	M1	M2	M3	M4
(a)				
Visual attributes				
Color intensity	4.74 ± 0.95a	4.73 ± 0.51b	3.82 ± 0.38b	3.54 ± 0.41b
Viscosity	4.09 ± 0.92	4.50 ± 0.59	4.30 ± 0.70	4.36 ± 0.76
Taste& Mouthfeel				
Sweetness	7.00 ± 0.24	6.60 ± 0.06	5.88 ± 0.39	6.96 ± 0.05
Sourness	0.41 ± 0.02	0.13 ± 0.08	0.58 ± 0.09	0.46 ± 0.07
Saltiness	0.40 ± 0.08	0.30 ± 0.07	0.30 ± 0.02	0.41 ± 0.03
Bitterness	0.00 ± 0.00	0.00 ± 0.00	0.00 ± 0.00	0.00 ± 0.00
Persistence	3.21 ± 0.97	3.35 ± 0.16	3.03 ± 0.63	3.15 ± 0.92
Astringency	0.12 ± 0.04	0.15 ± 0.03	0.09 ± 0.02	0.14 ± 0.03
Freshing sensation	0.08 ± 0.29	0.00 ± 0.00	0.00 ± 0.00	0.00 ± 0.00
Texture attributes				
Adhesiveness	4.37 ± 0.39	4.18 ± 0.65	4.65 ± 0.34	3.92 ± 0.24
Granularity	0.00 ± 0.00	0.00 ± 0.00	0.00 ± 0.00	0.00 ± 0.00
(b)				
Olfactory attributes				
Global odor intensity	6.60 ± 0.38	5.60 ± 0.30	5.35 ± 0.55	5.82 ± 0.21
Floral	4.66 ± 0.91	4.58 ± 0.95	4.62 ± 0.75	4.36 ± 0.47
Fruity	0.76 ± 0.12	0.93 ± 0.16	0.91 ± 0.26	1.22 ± 0.05
Warm	0.99 ± 0.15	0.94 ± 0.25	0.70 ± 0.14	1.37 ± 0.10
Aromatic	0.08 ± 0.02	0.00 ± 0.00	0.00 ± 0.00	0.00 ± 0.00
Vegetal	1.60 ± 0.51	1.40 ± 0.35	1.46 ± 0.41	1.02 ± 0.33
Chemical	0.60 ± 0.13b	0.17 ± 0.03^a,b^	0.00 ± 0.00a	0.03 ± 0.12a
Animal	0.00 ± 0.00	0.00 ± 0.00	0.00 ± 0.00	0.00 ± 0.00
Aroma attributes				
Aroma intensity	6.23 ± 0.93	5.83 ± 0.73	5.35 ± 0.21	6.28 ± 0.33
Floral	4.73 ± 0.36	4.40 ± 0.14	4.35 ± 0.48	4.65 ± 0.21
Fruity	0.91 ± 0.31	0.76 ± 0.08	0.69 ± 0.08	0.83 ± 0.12
Warm	2.85 ± 0.40	2.93 ± 0.28	2.27 ± 0.17	3.04 ± 0.07
Aromatic	0.25 ± 0.22	0.03 ± 0.13	0.10 ± 0.21	0.48 ± 0.08
Vegetal	1.24 ± 0.45	0.90 ± 0.28	0.99 ± 0.20	1.33 ± 0.25
Chemical	0.00 ± 0.00	0.00 ± 0.00	0.00 ± 0.00	0.00 ± 0.00
Animal	0.00 ± 0.00	0.00 ± 0.00	0.00 ± 0.00	0.00 ± 0.00

Note

M1: honey from non‐supplemented bees; M2: honey from supplemented bees with sugar; M3: honey from supplemented bees with sugar + vitamins+amino acids; M4: honey from supplemented bees with sugar + 3% of protein. Values within rows with different letters differ significantly (*p* < .05).

Finally, no significant differences (*p* < .05) were found for texture attributes (adhesiveness and granularity) for samples M1, M3, and M4 regarding the type of supplementation.

## 4 CONCLUSIONS

The results of the present study indicated that, regardless of any type of supplementation, all the honey obtained were in accordance with the international legislation in terms of physicochemical properties.

An analysis of the sensory characteristics showed that the control sample only presented significant differences for color and chemical odor attributes compared with honey from bees receiving supplementation.

Honey samples from bees receiving sugar paste + 3% proteins were classified first for the odor attributes fruity and warm. They also had higher values for aroma attributes like the intensity of aroma, warm, aromatic, and vegetal. The result reported in this study will be found useful by apiarists to help them understand the impact of supplementation of honeybee diets with sugar and proteins.

## CONFLICT OF INTEREST

There is no conflict of interest to declare.

## ETHICAL APPROVAL

The article does not contain any study with animals or human subjects.
